# Expectations of Continuous Vital Signs Monitoring for Recognizing Complications After Esophagectomy: Interview Study Among Nurses and Surgeons

**DOI:** 10.2196/22387

**Published:** 2021-02-12

**Authors:** Mathilde van Rossum, Jobbe Leenen, Feike Kingma, Martine Breteler, Richard van Hillegersberg, Jelle Ruurda, Ewout Kouwenhoven, Marc van Det, Misha Luyer, Grard Nieuwenhuijzen, Cor Kalkman, Hermie Hermens

**Affiliations:** 1 Department of Cardiovascular and Respiratory Physiology University of Twente Enschede Netherlands; 2 Department of Biomedical Signals and Systems University of Twente Enschede Netherlands; 3 Department of Anesthesiology University Medical Center Utrecht Utrecht Netherlands; 4 Department of Surgery Isala Zwolle Netherlands; 5 Connected Care Centre Isala Zwolle Netherlands; 6 Department of Surgery University Medical Center Utrecht Utrecht Netherlands; 7 Department of Surgery ZGT Hospital Almelo Netherlands; 8 Department of Surgery Catharina Hospital Eindhoven Netherlands

**Keywords:** telemedicine, physiological monitoring, vital signs, esophagectomy, postoperative complications

## Abstract

**Background:**

Patients undergoing esophagectomy are at serious risk of developing postoperative complications. To support early recognition of clinical deterioration, wireless sensor technologies that enable continuous vital signs monitoring in a ward setting are emerging.

**Objective:**

This study explored nurses’ and surgeons’ expectations of the potential effectiveness and impact of continuous wireless vital signs monitoring in patients admitted to the ward after esophagectomy.

**Methods:**

Semistructured interviews were conducted at 3 esophageal cancer centers in the Netherlands. In each center, 2 nurses and 2 surgeons were interviewed regarding their expectations of continuous vital signs monitoring for early recognition of complications after esophagectomy. Historical data of patient characteristics and clinical outcomes were collected in each center and presented to the local participants to support estimations on clinical outcome.

**Results:**

The majority of nurses and surgeons expected that continuous vital signs monitoring could contribute to the earlier recognition of deterioration and result in earlier treatment for postoperative complications, although the effective time gain would depend on patient and situational factors. Their expectations regarding the impact of potential earlier diagnosis on clinical outcomes varied. Nevertheless, most caregivers would consider implementing continuous monitoring in the surgical ward to support patient monitoring after esophagectomy.

**Conclusions:**

Caregivers expected that wireless vital signs monitoring would provide opportunities for early detection of postoperative complications in patients undergoing esophagectomy admitted to the ward and prevent sequelae under certain circumstances. As the technology matures, clinical outcome studies will be necessary to objectify these expectations and further investigate overall effects on patient outcome.

## Introduction

Surgical treatment of esophageal cancer is highly complex and associated with considerable postoperative morbidity. Although the centralization of care and introduction of minimally invasive surgery have improved clinical outcomes, complications still occur in approximately 60% of patients undergoing esophagectomy [1,2]. These postoperative complications contribute to mortality, prolonged hospitalization, and increased costs [3-6].

To prevent severe sequelae of complications after esophagectomy, early recognition of clinical deterioration is essential [7-9]. As complications are often preceded by detectable signs, such as atrial fibrillation or hemodynamic instability [10,11], patients are usually admitted to high-care units in the first days after surgery for close monitoring of vital signs (eg, heart rate, respiratory rate, blood pressure, body temperature) and other clinical markers. However, with the introduction of enhanced recovery pathways, patients tend to be transferred to surgical wards earlier [12,13]. Consequently, clinical signs of complications after esophagectomy present more often at the ward. Since the level of patient monitoring is typically lower in a ward setting, where vital signs are only measured a few times per day, this poses a risk of missing important early signs of deterioration.

As the market for wearable medical technologies grows, unobtrusive tools for wireless vital signs monitoring are emerging. By allowing continuous vital signs monitoring even while mobilizing, these technologies may aid early recognition of clinical deterioration in ward patients [14-18] and could therefore be of interest for patients undergoing esophagectomy. However, despite the potential promises, the technology is still immature, and further developments are needed to facilitate optimal implementation [19,20]. Furthermore, it is as of yet unclear how continuous monitoring should be integrated in current routines to promote effective care escalation. Accordingly, acceptance of the new technology and adoption by caregivers is uncertain, while this is crucial for effective implementation. Lastly, to date, there is still only scant evidence of the clinical value in specific patient populations [21]. Therefore, the aim of this study was to gain insight into nurses’ and surgeons’ expectations of the potential effectiveness and clinical impact of continuous vital signs monitoring in patients admitted to the surgical ward after esophagectomy.

## Methods

### Participants

We performed semistructured interviews with nurses and surgeons involved in the postoperative care of patients undergoing esophagectomy, which allowed thorough discussion of research topics from different perspectives. The study focused on surgical practice in the Netherlands, and interviewees were recruited from 3 Dutch high-volume centers for esophageal surgery (University Medical Center Utrecht, Catharina Hospital Eindhoven, ZGT Hospital Almelo). Purposive sampling [22] was applied to obtain a sample of care professionals with a high level of relevant expertise, aiming to promote in-depth discussion and informed judgements of the interview topics. Accordingly, in each participating center, the chair of the surgical (ward) team proposed candidates with the most knowledge and experience of postoperative monitoring of patients undergoing esophagectomy. Candidates were invited to participate in the study through email and gave written consent for the interview.

### Interview Setup

The interview setup and scheme (Multimedia Appendix 1) was developed by a group of 5 researchers and care professionals with expertise in the field of telemonitoring, clinical patient monitoring, esophageal surgery, and qualitative research. The interview included structured and open questions within 5 main themes. First, current approaches to patient monitoring after esophagectomy and factors influencing early recognition of postoperative complications were investigated. Subsequently, the participant’s expectations regarding the effectiveness and clinical impact of continuous vital signs monitoring were discussed. Last, considerations regarding the implementation of continuous monitoring were explored. As anastomotic leak and pneumonia are the most prevalent complications that can seriously affect clinical outcome in patients undergoing esophagectomy [1,3,23], these complications were used as case examples to discuss the topics and elicit concrete predictions.

Two pilot interviews were conducted—one with an experienced nurse (working experience: 9 years) and one with a surgeon (working experience: 2 years)—within one of the participating centers to verify whether questions were interpreted correctly and whether the research goals were obtained. Based on these test interviews, visual aids described below were added to further improve clarification of questions and structuration of the interview. Furthermore, the test interview led to the removal of questions regarding potential effect size, since the test participants indicated that the validity of such expert-based judgments would be questionable given the many uncertainties involved.

A researcher from an independent institute with a background in technical medicine and wireless patient monitoring performed all interviews in private workplaces within the hospital. The interviewer was guided by the interview scheme but was allowed to change the sequence of questions within main topics or to add questions for emerging topics. Rephrasing of questions and probing was used to encourage detailed answering. The interviews were audiotaped, and no notes were taken.

### Materials

The interviewer used visual aids for clarification of theoretical concepts and structured collection of information (Multimedia Appendix 1). The concept of continuous vital signs monitoring was introduced as the ability to constantly track heart rate, respiratory rate, body temperature, and oxygen saturation by means of unobtrusive wearable sensors that allow patient mobilization within the hospital. In addition, it was stated that automatic threshold alarms or (variations of) early warning scores could be integrated to assist detection of abnormalities.

To support and anchor estimations of potential clinical effects and minimize the possible influence of differences in preknowledge between participants, descriptive data of the local patient population were collected for each center and presented as the prior situation to the corresponding participants during the final part of the interview. Data included population characteristics, complication rates, and clinical outcome measures for all patients that underwent elective esophagectomy for nonrecurrent esophageal cancer between January 2015 and December 2016. All data were registered according to definitions by the Dutch Upper Gastrointestinal Cancer Audit [6,24] and collected prior to the interviews. Multimedia Appendix 2 summarizes the baseline characteristics of the pooled patient populations of the 3 participating centers.

### Analysis

The interviewer transcribed the interview recordings. Next, all transcripts were coded using Atlas.ti software (version 8.3.2; Atlas.ti Scientific Software Development) for content analysis [25]. Coding was performed independently by the interviewer and a second researcher with expertise in nursing and wireless patient monitoring. In this process, content was categorized according to the predefined interview topics, after which the results of structured questions were summarized and emerging themes within categories were coded. Codes were refined as analysis progressed and added when new themes emerged. Any discrepancies in coding by the 2 researchers were mutually discussed to obtain consensus for all codes and themes. The transcripts were not returned to the participants for correction to avoid censoring, and study findings were member checked after completion of the analysis. To evaluate the level of data saturation that was obtained, we assessed the number of new themes that were elicited across the inclusion of participants. In addition, we explored the number of themes mentioned exclusively by either nurses or surgeons or by participants of 1 center only. The results were reported following the Standards for Reporting Qualitative Research guidelines [26].

## Results

### Participants

All candidates that were invited for the interview participated in the study. The recruited nurses (n=6) had a median working experience of 7.5 years (range 2-25 years), of which they worked 4 years (range 1-25 years) with patients undergoing esophagectomy. The participating surgeons (n=6) had a median working experience of 11 years (range 6-21 years) in upper gastrointestinal surgery. Interviews had an average duration of 44 minutes (range 25-63 minutes).

### Data Saturation

Content analysis resulted in identification of 40 themes (Multimedia Appendix 3), of which 14 themes were described by participants in 2 centers and 25 themes were discussed in all included centers. In each center, at least 75% of all themes were described by at least one of the participants. In total, 85% of all themes were described by both nurses and surgeons. Analysis of the interviews from the last included participants did not result in elicitation of new themes (Multimedia Appendix 3); hence, sufficient data saturation was assumed.

### Current Monitoring Routine

Current protocols for patient monitoring during ward stay were similar among the 3 hospitals. Typically, a physician visited the patient during daily rounds and performed physical examination on indication. Chest radiography, blood tests of infection parameters, and drain amylase tests were performed daily in the first days after surgery. Each hospital used an early warning score system (similar to the Modified or National Early Warning Score [27,28]) to evaluate the patient’s status 3 to 4 times per day. As part of these early warning scores, standard vital sign measurements of heart rate, respiratory rate, blood pressure, oxygen saturation, and temperature were performed. This set was complemented by routine measurements of urine output and evaluation of mental status. However, participants of one hospital described that urine output and mental status were not assessed routinely for each patient but specifically in patients with suspected instability.

In case deterioration was suspected based on routine measurements and subjective nurse observations, additional physical examination, vital signs measurements, blood tests, or diagnostic imaging were performed to confirm findings and for further diagnosis. However, the approach of diagnostic confirmation seemed to vary between hospitals, as participants from one hospital promoted early activation of diagnostic imaging, while other participants advocated a wait-and-see policy to prevent overdiagnosis.

### Early Recognition of Complications

All participants underlined that early recognition of complications is important for rapid recovery and minimization of adverse clinical outcomes. Furthermore, all participants were confident that the current monitoring routine supports early complication recognition but recognized that the time to identification and treatment of complications depends on various factors.

The majority of participants reported that signs of anastomotic leak and pneumonia typically present first in vital signs measurements and subjective nurse observations (Multimedia Appendix 4). In a later phase, abnormalities often present in lab tests and physical examinations, followed by medical imaging. However, several participants pointed out that the presentation of clinical deterioration varies per patient and complication type. As one nurse explained:

The presentation of a complication differs between patients. Some patients are able to compensate for a long time, while other patients deteriorate immediately.

Participant 3

Participants noted that clinical deterioration is not always visible in an early stage or for a mild degree of complications, where physiology is still unaffected or impairment is too small to be captured by routine observations or diagnostic tests. Furthermore, compensatory mechanisms or medication may suppress signs of deterioration. As such, abnormalities may remain undetected.

Conversely, abnormal diagnostic test results or physical symptoms, for example, tachycardia, could relate to various complication types, which hampers differentiation in an early phase. Moreover, abnormalities can be caused by the surgical stress response, comorbidities, or normal variations. For these reasons, identification of deterioration relies on the combination of subjective observations and diagnostic tests. Accordingly, caregivers often wait to see whether the observed abnormalities persist or present in other diagnostic tests before acknowledging a (potential) complication. Last, half of the participating surgeons mentioned that routine test results are often assessed statically according to standard thresholds, while temporal changes are more indicative of deterioration.

Participants explained that late detection of clinical deterioration can be caused by incomplete or delayed routine examinations. Nurse observations and vital signs measurements may be skipped or postponed if the patient appears stable, in particular when workload is high. Additionally, vital signs are not always measured in patients who are asleep to avoid sleep deprivation. Lastly, the interval between the onset of deterioration and evaluation of test results depends on the timing of routine measurements and clinical rounds, which leads to variable response times.

A total of 6 participants mentioned that the level of expertise of the treating physician and nurse influences how fast deterioration is recognized and acted upon, as this impacts the ability to observe and interpret physical signs and identify abnormalities in diagnostic results. This mainly concerns weekend, evening, and night shifts, which are typically occupied by less experienced staff.

### Effectiveness of Continuous Vital Signs Monitoring

**Figure 1:**
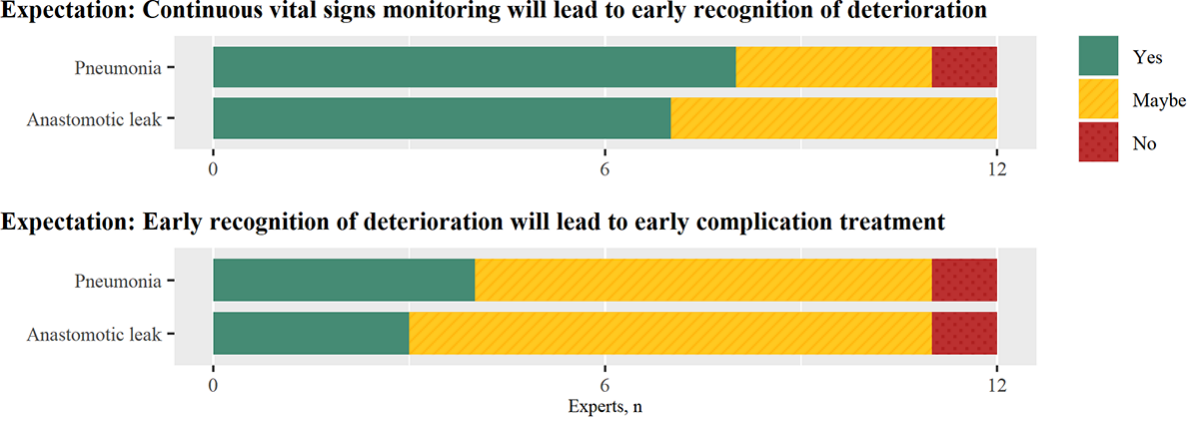
Experts’ expectations of the effectiveness of continuous vital signs monitoring.

The majority of participants expected that continuous vital signs monitoring could support early recognition of deterioration related to anastomotic leak and pneumonia (Figure 1). A total of 6 participants estimated a maximal time gain of 1 to 8 hours, deduced from the fact that continuous availability of data can facilitate direct notification of (acute) abnormalities and hence fill the gap between current intermittent measurements, which are typically obtained every 8 hours. Conversely, 5 participants argued that the time gain could be higher and might reach 12 to 48 hours, mainly supported by the increased ability to identify time trends or abnormal patterns. As one surgeon described:

With the availability of continuous data, we can better observe trends, which are more important than spot-checks….These patterns influence our judgement of the patient’s status.

Participant 4

This can be of particular benefit for patients with slowly developing complications or in cases where deterioration is not suspected due to unspecific or absent physical signs. Lastly, 3 participants also described that it is likely that continuous monitoring promotes early identification by increasing the awareness of potential abnormalities. Next to pneumonia and anastomotic leak, participants mentioned that continuous monitoring could contribute to early detection of arrhythmias, such as atrial fibrillation, infections, and severe acute events, such as pulmonary embolism and myocardial infarction.

The participants who were more doubtful about the ability to recognize deterioration early mostly ascribed this to the limited sensitivity and specificity of vital signs measurements and the importance of full clinical assessment. A nurse stated:

These numbers don’t tell the whole story.

Participant 2

Furthermore, it was argued that early warning does not just rely on vital signs, since first signs of complications could be observed in other measurements at the same time or even earlier depending on presentation (Multimedia Appendix 4). Last, several participants stated that it is unlikely that deterioration can be identified earlier, as current routines are already effective and caregivers are constantly alert to potential complications.

Most participants expected that early notification of deterioration effected by continuous vital signs monitoring would lead to earlier treatment of the underlying complication in (a subset of) patients (Figure 1). Participants pointed out that continuous monitoring would also promote earlier activation of therapy by increasing the certainty that abnormalities persist or providing an objective description of the patient status that could be used to justify escalation of care. The overall effect on time to treatment might, however, be limited, as clinical progress or diagnostic confirmation is often awaited first. A nurse explained:

There are cases where we have to wait and follow-up the measurements. Then we can identify whether the patient is indeed deteriorating or stabilizes.

Participant 2

Six participants stated that the implementation of active alarms is crucial for effective monitoring, as these could raise the awareness of abnormal vital signs. One of the surgeons mentioned:

Alarms will trigger caregivers to actively search for abnormalities….I think this will specifically improve the continuity of early recognition.

Participant 11

By supporting identification of abnormalities, automated alarms can reduce nurse workload and minimize the dependency on nurse expertise. However, it was also mentioned that alarm-based response systems may have unintended consequences, such as neglecting subjective patient observation, which should be prevented, as this is important for adequate patient assessment. Furthermore, it is crucial that notifications are given at the right time and that the number of false alerts is minimal to prevent alarm fatigue. A total of 3 surgeons stated it would be valuable to complement the static assessment of vital sign values by automatic trend detection.

Most participants mentioned that implementation of continuous monitoring requires training for nurses and physicians in the practical use of the monitoring system or interpretation of continuous vital signs. In addition, 10 participants underlined the need for a clear protocol that defines the responsibilities of clinical staff and describes when and how to act in case of vital sign abnormalities. However, it was also noted that it is first needed to gain more insight into patterns of deterioration that require escalation of care and that it would take time to find out and establish effective monitoring routines.

### Impact of Continuous Vital Signs Monitoring

**Figure 2:**
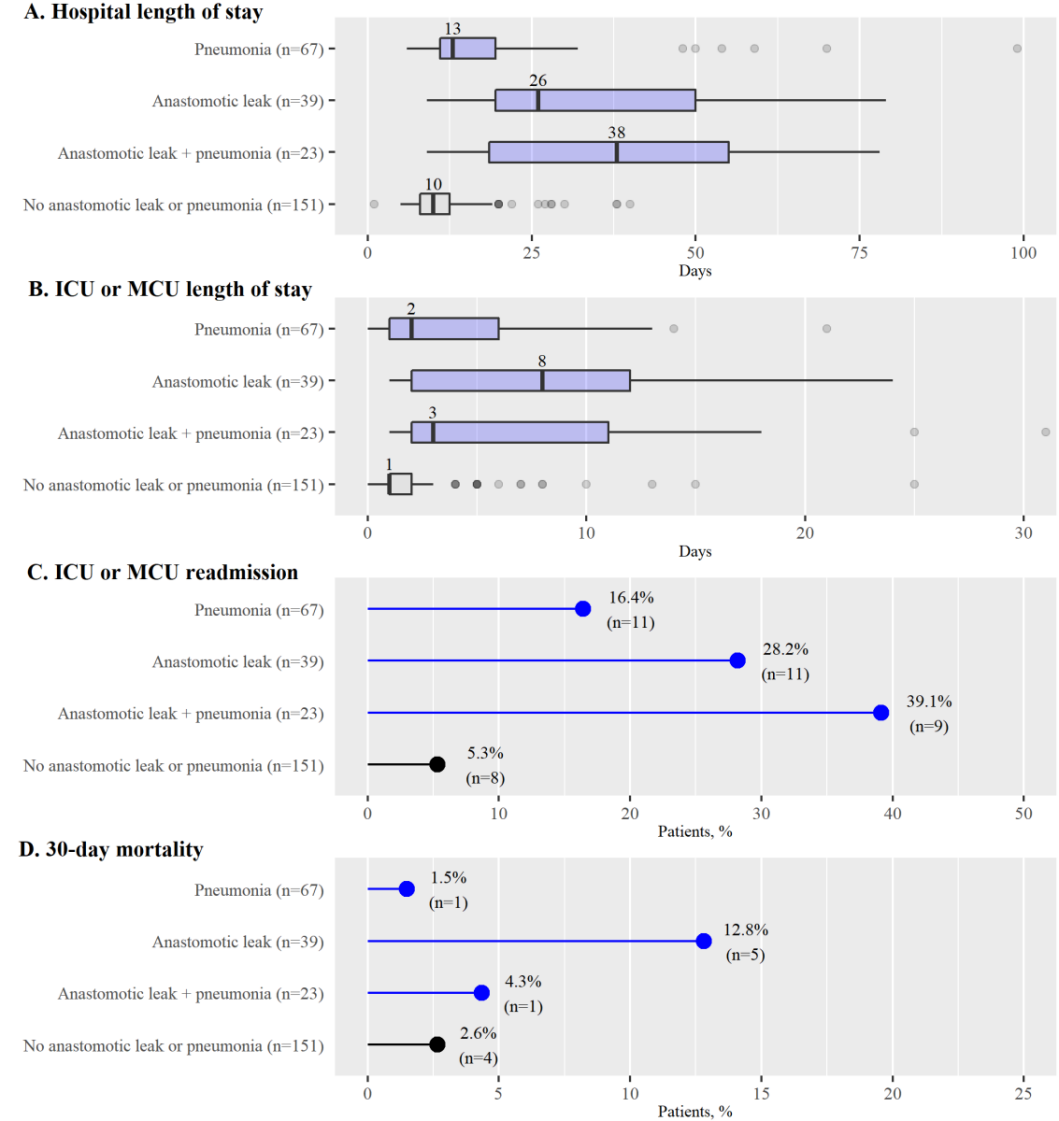
Clinical outcome of patients undergoing esophagectomy. Outcomes are reported for the pooled patient population that underwent elective esophagectomy between 2015 and 2016 in one of the 3 participating centers (n=280). Subgroups reflect patients with or without pneumonia, anastomotic leak, or both within 30 days after surgery. ICU: intensive care unit; MCU: medium care unit.

**Figure 3:**
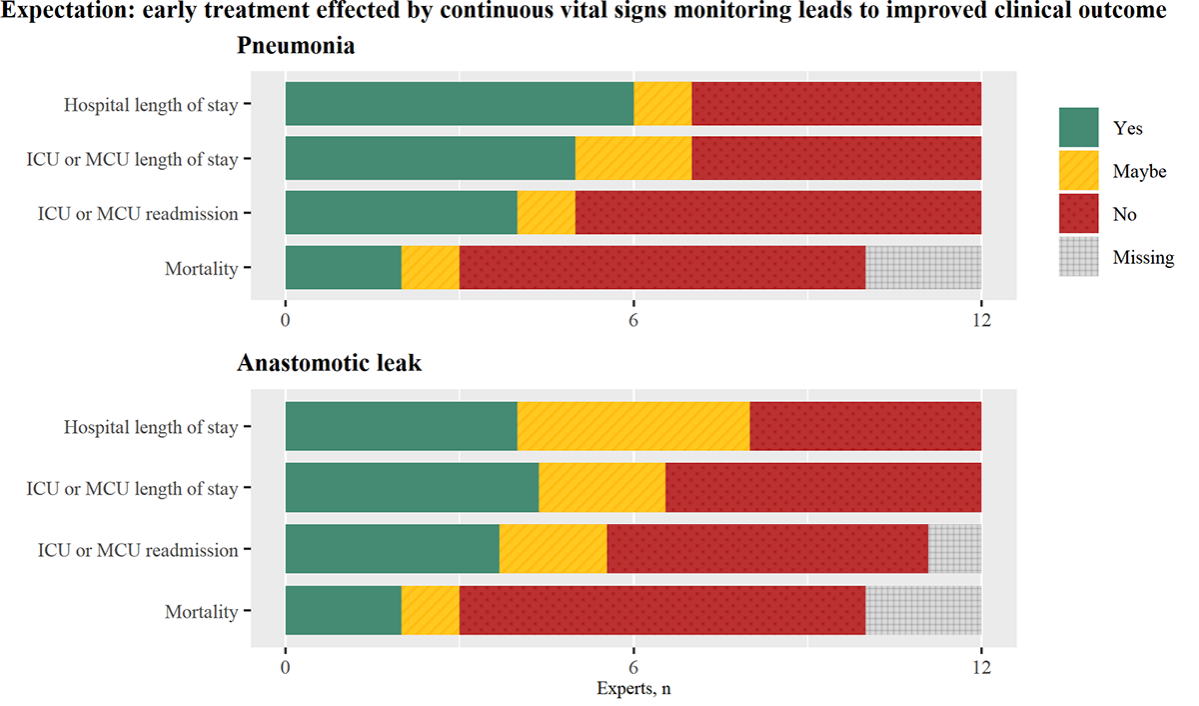
Experts’ expectations of the improvement of clinical outcome measures. ICU: intensive care unit; MCU: medium care unit.

The combined data from the 3 participating hospitals (Figure 2) showed that patients who developed postoperative pneumonia, anastomotic leak, or both had a considerably longer length of hospital stay and increased risk of intensive care unit (ICU) or medium care unit (MCU) readmission. Furthermore, the data suggest that anastomotic leak strongly increases mortality. Overall, a minority of participants expected that early recognition and treatment of pneumonia and anastomotic leak effected by continuous monitoring would improve these outcome measures, as shown in Figure 3.

Participants who expected a reduced hospital and ICU or MCU length of stay assigned this either to a shortened recovery and treatment period or to earlier onset and hence completion of the treatment period. Improvement in ICU or MCU readmission rate and mortality was attributed to a potential reduction in complication severity. Two participants stated that early recognition is of the highest value in patients with mild complications, as prevention of further deterioration would still be relatively easy. In contrast, 2 other participants expected the most impact in cases of severe complications because there would be more room to reduce the degree of illness. Furthermore, it was mentioned that the largest benefits could be expected in patients with a poor preoperative condition, as these have a higher risk of severe deterioration.

A total of 5 participants mentioned that the time gain that could be obtained with continuous monitoring is insufficient for notable improvement of clinical outcome. One nurse stated:

The hours that we could possibly gain on top of our current protocol are not enough to impact the progress or severity of the complication.

Participant 6

Participants who were doubtful indicated that the minimal time gain required for significant reduction of adverse effects caused by complications would range from 12 to 48 hours. Lastly, it was pointed out that adverse effects of some complications cannot be minimized at all because early onset of treatment does not reduce the duration of hospitalization or change patient outcomes.

### Considerations for Implementation

Taking all potential effects into account, 10 participants would consider implementing continuous monitoring on their ward for early detection of deterioration. Most of these participants (n=6) would monitor all patients undergoing esophagectomy, while others preferred preselection of older patients (n=1) or patients with a poor preoperative condition (n=1). Several participants considered applying continuous monitoring only during the first days of ward stay (n=2) or in case of nurse concerns (n=1).

The main argument against implementation included the expectation that continuous monitoring would not bring sufficient benefit on top of current monitoring protocols due to limited clinical effects. Furthermore, 5 participants mentioned that improvement in patient monitoring is becoming less relevant, as the prevalence and severity of complications is reducing over the years. A surgeon said:

Patients have a lower risk of developing complications than a few years ago.…Also, the effects of complications are less severe. So, we now have more room to await clinical progress.

Participant 5

Participants described additional risks and benefits related to patient experience, nurse workload, and financial consequences but were divided on these topics. Several participants suspected that continuous monitoring would create a feeling of safety for patients. On the other hand, other participants expected worry related to false alarms and the feeling of being at risk. Furthermore, it was noted that the sensor placement and potential overdiagnosis could increase patient burden.

While most participants expected a reduction of nurse workload from (partial) automated vital signs measurement, others warned of increased workload related to vital sign interpretation and management of alarms. Moreover, 3 nurses suspected that the implementation of continuous monitoring would also create increased expectations of the level of care. One of these nurses stated:

In case you monitor patients continuously, you will also need to be able to provide continuous response.

Participant 6

However, they feared that this level of care could not be met, as the available time and expertise of the ward nurse staff is currently insufficient.

Lastly, participants reported that cost might be saved as a result of reduced hospital length of stay and reduced intensive care readmissions but also noted that expenditures might increase due to the costs of monitoring systems.

## Discussion

### Principal Findings

This study identified perceptions of surgeons and nurses on the potential clinical effects of continuous vital signs monitoring by means of wearable sensors in patients admitted to the ward after esophagectomy. Caregivers suspected that continuous vital signs monitoring could promote early recognition of clinical deterioration in this population and setting and contribute to early treatment of prevalent complications. However, there were varying expectations regarding whether continuous monitoring would lead to notable improvements in hospital length of stay, ICU readmission, and mortality. Despite an as of yet uncertain clinical impact, most caregivers are positive toward future implementation of continuous vital signs monitoring to support patient monitoring in the surgical ward, provided that their concerns are adequately addressed.

### Previous Studies

The perioperative management of patients undergoing esophagectomy has evolved over the years, and there is growing attention to the importance of early complication recognition [8,11]. According to current study results, however, there is still room to improve early detection of complications in a ward setting, which conforms to findings of previous studies [17,29]. Vital signs and related early warning systems have been found to be good predictors of ICU transfer, cardiac arrest, and mortality [16,30,31]. Therefore, there are high expectations of the potential value of continuous wireless vital signs monitoring, which allow more accurate and constant risk evaluation [14,32,33].

Although evidence is still scarce, previous studies have described how continuous vital signs monitoring using wearable sensors could promote early identification of patient deterioration in a ward setting [21,34-37], which was also expected by these study participants. Furthermore, wireless monitoring has been proposed as a promising aid in other settings, for example, to assist in- or out-of-hospital monitoring of isolated patients during the current COVID-19 pandemic or surgical patients with restricted access to medical services [38].

However, previous studies have reported variable effects of continuous monitoring on patient outcomes and cost efficiency [21,36], which is in line with the mixed expectations regarding clinical impact found in our study. Part of this inconclusive evidence can be explained by the fact that most studies so far have included small or heterogeneous study populations and used different monitoring strategies. Furthermore, continuous monitoring has often not been implemented at its full potential, restricted by the constraints of current available technology or limited compliance to the monitoring or response protocols. Moreover, the monitoring protocols have often adopted a classical approach to vital signs assessment based on static vital signs levels. However, as described by current participants and in previous research [39], continuous and automated monitoring creates additional opportunities for trend evaluation and integration with context data, which may improve identification of deterioration. Accordingly, further investigation of adequate methods for trend-based and personalized assessment of vital signs data is encouraged.

On the other hand, these discrepant expectations regarding the possible clinical impact of continuous monitoring may also represent the complexity of managing postoperative surgical complications, where the ability to minimize adverse outcomes depends not only on early detection and treatment but also on the effects of the selected interventions. As the implementation of continuous monitoring introduces a risk of alarm fatigue and patient discomfort [21], studies that identify patients that would benefit most from continuous remote monitoring and early treatment are desired. Correspondingly, our study participants underlined the importance of establishing feasible but effective protocols for escalation of care. Furthermore, the responsibilities of caregivers and work processes should be adjusted with care to encourage adoption by caregivers and promote the effective implementation of continuous monitoring. The results of this interview study indicate that even if vital signs monitoring triggers early suspicion of deterioration, clinical observation as well as complementary diagnostic tests are imperative for the correct interpretation and actual diagnosis of complications. However, the introduction of continuous monitoring could also lead to overreliance in monitoring technology [29,33]. Therefore, careful implementation is required to balance the risks of missed events and overdiagnosis.

### Strengths and Limitations

The qualitative design of this study allowed us to obtain estimations from professionals caring for patients undergoing esophagectomy, a highly complex surgical procedure associated with considerable risk, regarding the effectiveness of continuous monitoring technology. By using expert elicitation, the potential of continuous monitoring in the postoperative setting could be evaluated in the early development phase, where technology is evolving rapidly and the reliability, accuracy, and usability of these systems still need to be demonstrated [14]. Another advantage of this theoretical approach is that the results were not affected by the local implementation of technology or compliance of patients and caregivers, which could distort evaluation in clinical studies [21]. Furthermore, the interviews allowed stepwise investigation of individual components of the monitoring and response chain, which is challenging in a clinical setting.

However, as reflected by current findings, there are many patient-related or situational factors that might influence the effectiveness and impact of continuous patient monitoring and also challenge theoretical effect estimation. To promote the validity of estimates from caregivers, we therefore focused on a highly specific patient population and used case examples to minimize uncertainty. Furthermore, we purposely included only experienced caregivers from specialized centers within a single country to participate in the study to compose a homogeneous group of experts (ie, information-rich cases). Last, historical data of the local patient population were used to describe current clinical outcomes and create a consistent anchor point for effect evaluation. Nevertheless, current estimations can only be used hypothetically, and the overall impact on clinical outcome measures requires confirmation in clinical practice.

This study included surgeons and well as nurses from 3 centers. This allowed us to investigate topics and viewpoints from both the nursing and surgical professions and possible local perspectives within the Netherlands. According to national registries, these high-volume centers were responsible for 17% of all esophagectomies performed in the Netherlands in 2015 to 2016, and they reported similar population characteristics as those described for the national population [40]. Furthermore, except for some variation in the frequency and type of routine vital signs measurement, the overall clinical routines and escalation protocols were largely comparable between centers. Therefore, it is likely that the research sample is representative of the situation in the Netherlands. Although we conducted a limited number of interviews, viewpoints of participants or themes that were described by participants did not vary considerably within or between centers or between nurses and surgeons. In addition, as no new themes emerged from the interviews of the last included participants, sufficient saturation was assumed. Still, since the patient population and clinical routines may differ in other centers or countries, careful translation of findings for other settings is required.

### Implications

As our study reflects that caregivers see opportunities to improve postoperative care after esophagectomy using wireless continuous vital signs monitoring, future studies that verify this potential in a ward setting are encouraged. By explicating factors that define the need for and ability of early complication recognition, current results may guide stepwise investigation of the effective time gain and corresponding clinical and economic effects of various monitoring strategies. As such, the optimal implementation of continuous wireless vital signs monitoring can be further evaluated as the technology matures.

### Conclusions

Despite routine monitoring, identification of postoperative complications in patients undergoing esophagectomy admitted to the ward may be delayed due to limited frequency and diagnostic value of diagnostic measurements and the variable experience and skills of clinical staff. Surgeons and nurses expect that continuous vital signs monitoring by means of emerging wearable sensor technology would provide opportunities for early detection of clinical deterioration, which could promote rapid complication treatment. However, the effective time gain and impact on clinical outcome are yet uncertain and depend on patient and situational factors. Further investigation of the overall benefits and risks and optimal implementation of continuous vital signs monitoring is desired as technology matures.

## Appendix

Multimedia Appendix 1 Interview guide.

Multimedia Appendix 2 Baseline characteristics of patient population undergoing esophagectomy.

Multimedia Appendix 3 Themes elicited during context analysis.

Multimedia Appendix 4 Typical order of early signs observed for pneumonia and anastomotic leak.

## Abbreviations

ICU: intensive care unit
MCU: medium care unit
