# Supplementary material for: Expectations of Continuous Vital Signs Monitoring for Recognizing Complications After Esophagectomy: Interview Study Among Nurses and Surgeons
Source: JMIR Perioper Med. 2021 Feb 12;4(1):e22387. doi: 10.2196/22387 (PMC7910120; doi:10.2196/22387)
Supplement: Multimedia Appendix 1 [file periop_v4i1e22387_app1.pdf]

Interview scheme

Instruction

- The interview scheme describes main interview topics and corresponding question topics. The introduction of topics or visual aids is described in italic. The checkboxes (□) list items that should be discussed with each participant as part of the topic exploration. If these items were not addressed by participants themselves as response to the open question, these were introduced later by the interviewer.
- The interviewer should introduce main topics and the corresponding visual aids in the order as described. The sequence of questions within main topics can be changed, and questions for emerging topic can be added. Rephrasing of questions and probing can be used to encourage detailed answering.

Main topic	Topics of questions
Participants' background	*Introduction of interview goals and set-up*<br><br>- Participant's position in hospital<br>- Years of working experience
Current monitoring routine	*Introduction of scope: participants undergoing esophagectomy admitted to ward.*<br>*Introduction of common types of routine measurements:* ***visual aid 1***<br><br>- Type and frequency of measurements in patient monitoring routine in clinical ward<br>☐ Subjective nurse observation<br>☐ Physician round<br>☐ Physical examination<br>☐ Vital signs<br>☐ Lab tests<br>☐ Medical imaging<br>- Situations where monitoring routine is performed differently<br>- First actions in case of abnormalities<br>☐ Involve other caregivers<br>☐ Additional diagnostic actions<br>☐ Emergency intervention team

<table>
<tr><td>Early recognition of complications</td><td>- Order of presentation of abnormalities during complication development: pneumonia + anastomotic leak
    ☐ Subjective nurse observation
    ☐ Physician round
    ☐ Physical examination
    ☐ Vital signs
    ☐ Lab tests
    ☐ Medical imaging
- Ability to detect complications in early phase in current practice
- Factors influencing the time to detect complications
    ☐ Measurement type
    ☐ Care professionals
- Consequences of late detection of complications
    ☐ Patient outcome
    ☐ Clinical trajectory of patient</td></tr>
<tr><td>Effectiveness of continuous vital signs monitoring</td><td>Introduction of theoretical mechanism of patient monitoring: visual aid 2
Introduction of telemonitoring concept: visual aid 3

- Expectation: continuous monitoring will lead to earlier detection of deterioration: pneumonia + anastomotic leak
- Factors influencing or explaining the expected possibility to improve the time to detect of complications by introducing continuous monitoring
- Expected time gain in detection of complications
- Complication types that can be detected earlier using continuous monitoring
- Expectation: the expected time gain to detect complication will lead to earlier treatment: pneumonia + anastomotic leak
- Factors influencing or explaining the expected possibility to improve time to treat complications by introducing continuous monitoring
- Prerequisites for early detection and treatment
    ☐ Care protocol
    ☐ Tasks and responsibilities of care professionals</td></tr>
</table>

Impact on clinical outcome	*Introduction of demographic data and clinical outcome data of hospital:* ***patient data handout**** – Expectation: the expected time gain to detect and treat complications will improve clinical outcome: pneumonia + anastomotic leak ☐ Length of hospital stay ☐ Length of ICU/MCU stay ☐ ICU/MCU readmission rate ☐ Mortality rate – Factors influencing the possibility to improve clinical outcome by introducing continuous monitoring
Considerations for implementation	– Risks and benefits for stakeholders: ☐ Patient ☐ Nurse ☐ Physician ☐ Hospital ☐ Health insurance company – Overall interest to implement continuous monitoring in ward – Patient where continuous monitoring would be indicated – Factors influencing the overall interest in continuous monitoring

* Handout with data of the population characteristics, complication rates, and clinical outcome measures for all patients that underwent elective esophagectomy for non-recurrent esophageal cancer between January 2015 and December 2016. Only data obtained in the participant's hospital is shown to the participant.

Visual aids

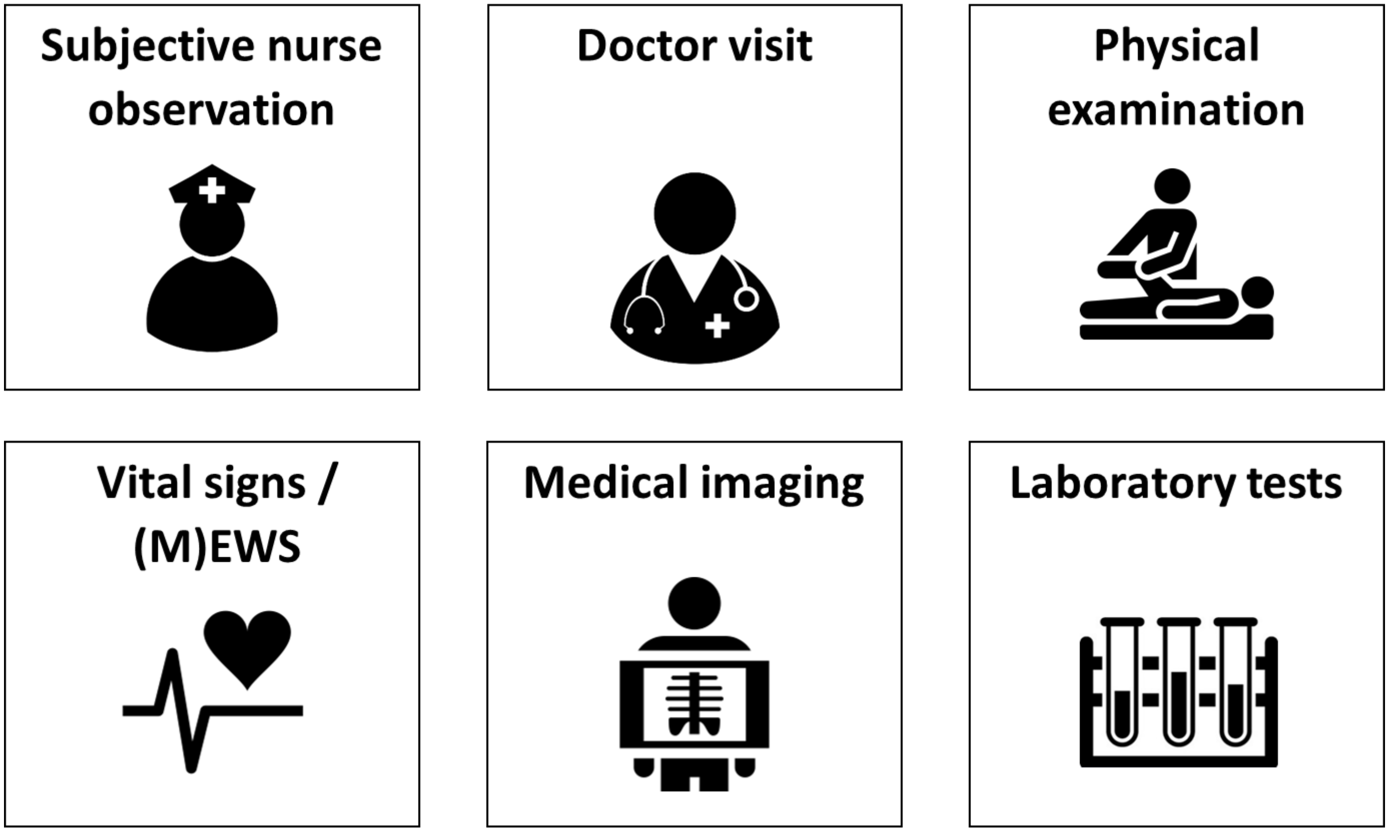

Visual aid 1. Visual aid used to support identification of measurements performed in current patient monitoring routine

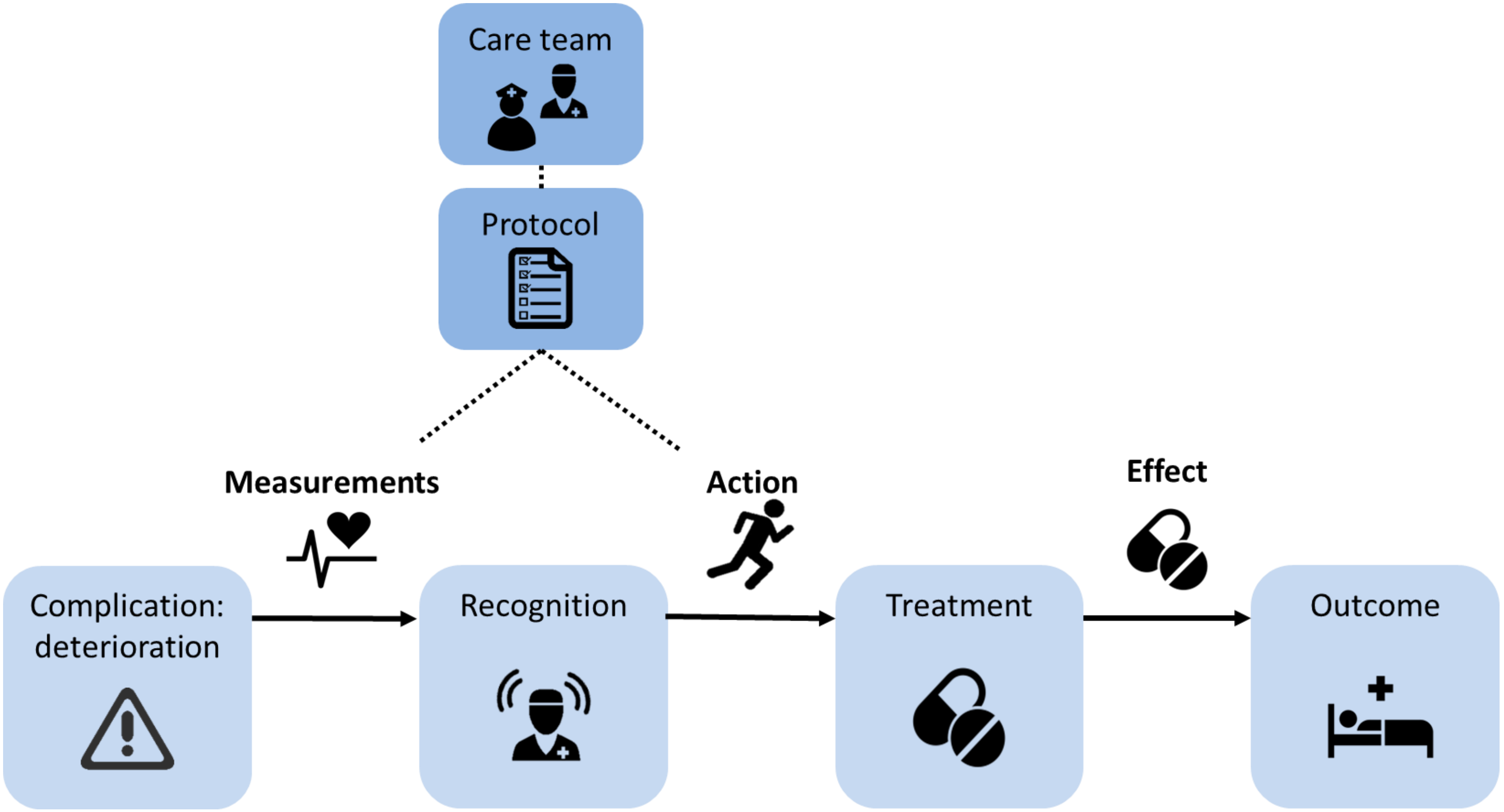

Visual aid 2. Visual aid to clarify the theoretical mechanism of patient monitoring

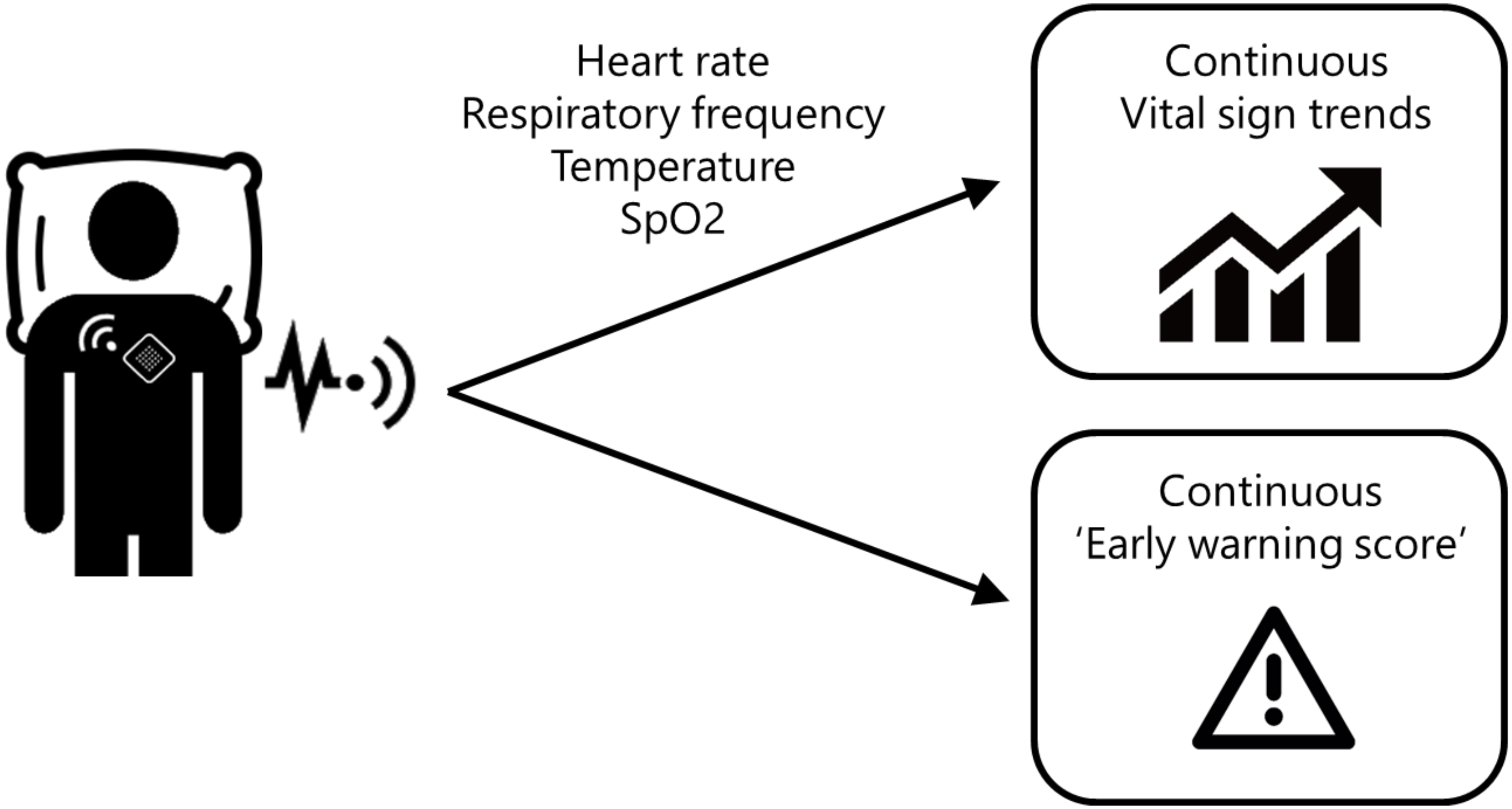

Visual aid 3. Visual aid to clarify concept of continuous vital signs monitoring
