# Supplementary material for: Expectations of Continuous Vital Signs Monitoring for Recognizing Complications After Esophagectomy: Interview Study Among Nurses and Surgeons
Source: JMIR Perioper Med. 2021 Feb 12;4(1):e22387. doi: 10.2196/22387 (PMC7910120; doi:10.2196/22387)
Supplement: Multimedia Appendix 2 [file periop_v4i1e22387_app2.pdf]

Baseline characteristics of patient population undergoing esophagectomy[1]

Population characteristic	Classification	Result
Number of patients	N.A.	280
Male	N.A.	224 (80%)
Age at time of surgery (years)	N.A.	64 (8.6)
BMI (kg /m2)	N.A.	25.8 (4.1)
ASA classification	I	36 (12.9%)
	II	189 (67.5%)
	III	55 (19.6%)
Comorbidities	Any comorbidity	203 (72.5%)
	Cardiac comorbidity	56 (23.9%)
	Vascular comorbidity	108 (46.2%)
	Pulmonal comorbidity	64 (27.4%)
	Diabetes comorbidity	34 (14.5%)
	Urological comorbidity	23 (9.8%)
	Thrombo-embolic history	12 (5.1%)
Surgery type	Open	19 (6.8%)
	Minimally-invasive	261 (93.2%)
Surgical approach	Transhiatal	15 (5.4%)
	Transthoracic	265 (94.6%)
Anastomosis location	Cervical	118 (42.1%)
	Thoracic	162 (57.9%)
Neoadjuvant therapy	Chemotherapy	8 (3.0%)
	Chemoradiotherapy	247 (92.9%)
	Radiotherapy	1 (0.4%)
	No therapy	24 (8.6%)
Peroperative complication	N.A.	13 (4.6%)
Postoperative complication (within 30 days after surgery)	Pneumonia	90 (32.1)
	Anastomotic leak	62 (22.1)
	Other complication	122 (43.6)
	No complication	85 (30.4)

Values reflect the number of patients (% of total) or mean value (standard deviation). N.A.: not applicable; ASA: American Society of Anesthesiologists.

[1]Pooled population characteristics of patients undergoing elective esophagectomy between 2015 and 2016 in one of the three centers participating in this study.
