# Supplementary material for: Expectations of Continuous Vital Signs Monitoring for Recognizing Complications After Esophagectomy: Interview Study Among Nurses and Surgeons
Source: JMIR Perioper Med. 2021 Feb 12;4(1):e22387. doi: 10.2196/22387 (PMC7910120; doi:10.2196/22387)
Supplement: Multimedia Appendix 3 [file periop_v4i1e22387_app3.pdf]

Themes elicited during context analysis

Main topic	Theme	Specification of theme
Current monitoring routine	Caregivers policy towards speed of activating further diagnostics	The policy towards the use of diagnostic imaging differs between hospitals, as caregivers either promote early activation or a wait-and-see policy
Early recognition of complications	Variable presentation of deterioration	The presentation of deterioration differs per patient
	Absence of early signs	Signs of deterioration are not visible in early phase of complications or in current routine measurements
	Limited specificity of routine measurements	Abnormal results of individual routine measurements are not enough to confirm complication
	Expertise level	Abnormalities may not be recognized or acted upon by less experienced clinicians or nurses
	Incomplete or delayed measurements	Early signs may be missed or delayed due to delayed or incomplete measurements
	Importance of trends	Trends in diagnostic test results are important to identify clinical deterioration or to recognize specific complications
Effectiveness of continuous vital signs monitoring	Limited sensitivity and specificity of vital signs	Vital signs have limited sensitivity and specificity for detection of specific complications, as abnormal vital sign levels may be caused by other factors and because complications do not always present with abnormalities in vital signs
	Effectivity of current patient monitoring approach	Current patient monitoring approach already promotes fast detection and response towards potential complications, and there is little room for further improvement
	Early signs already seen in other measurements	Early signs of deterioration observed in vital signs are preceded or accompanied by signs of deterioration in other measurements or clinical observations
	Continuity of measurements can fill current time gap	Early recognition of deterioration is expected because of the continuity of measuring, such that abnormalities are seen earlier as compared to current intermittent vital sign measurements
	Improved ability to identify trends	Early recognition of deterioration is expected because of the improved ability to identify trends
	Increased awareness	Early recognition of deterioration is expected because of the increased awareness to vital sign abnormalities, caused by continuous measurements or active alarms
	Objective criteria for care escalation	Early action is promoted by providing an objective description of the patient status that can be used to justify escalation of care
	Confirmation of deterioration	Early action is promoted by providing extra confirmation of event/deterioration
	Clinical progress is awaited	Early recognition of deterioration will not lead to early treatment because clinical progress is awaited
	Diagnostic confirmation is awaited	Early recognition of deterioration will not lead to early treatment because diagnostic confirmation is awaited
	Need for escalation protocol	It should be clearly described what vital signs values are abnormal and which action is required

Themes elicited during context analysis (continued)

Main topic	Theme	Specification of theme
Effectiveness of continuous vital signs monitoring	Need for training in use of technology	Clinical staff should be trained in how to use the monitoring system and activate measurements
	Need for training in interpretation of vital signs	Clinical staff should be trained in how to interpret the measurements
	Need for automated alarms	Automated alarms / notifications are important for effective monitoring
	Need for trend assessment	(Automated) trend assessment is desired to support identification of deterioration in vital signs
	Need to retain subjective observations	The subjective evaluation by clinicians is important for adequate patient assessment, and should not be neglected
	Optimal implementation requires more insight	Experience and further research is required to find and establish effective monitoring routines
Impact of continuous vital signs monitoring	Treatment period cannot be reduced	Complications require a certain treatment trajectory, which cannot be reduced with early recognition
	Insufficient time gain to improve clinical outcome	The expected potential time gain is not enough to improve clinical outcome measures
	Clinical outcome cannot be changed with early treatment	Clinical outcome of a given complication cannot be changed at all with early recognition or treatment
	Shifted treatment period	Early onset of treatment results in early completion, which reduced length of hospital stay
	Reduction of complication severity	Improvement in clinical outcome effected by reduced complication severity
	Faster recovery	Improvement in clinical outcome effected by faster recovery
	Impact depends on situation	The clinical impact affected by complication severity and patient characteristics
Considerations for implementation	Relevance dependent on complication number and severity	The clinical relevance of continuous monitoring depend on the number and severity of complications
	Nurse workload	Risk of increased nurse workload related to vital signs interpretation and response towards abnormalities
	Expectations towards level of care	Risk that the implementation of continuous monitoring will create increased expectations towards the level of care, assuming continuous surveillance and response
	Overdiagnosis or overtreatment	Risk of overdiagnosis and overtreatment
	Patient comfort	Risk of impaired patient comfort
	Patient experience	Effect on patient’s experience or feeling of safety
	Financial consequences	Costs or financial gains
	Efficiency	Efficiency related to remote access and automation
	Alarm fatigue	Risk of high (false) alarm rates and associated alarm fatigue

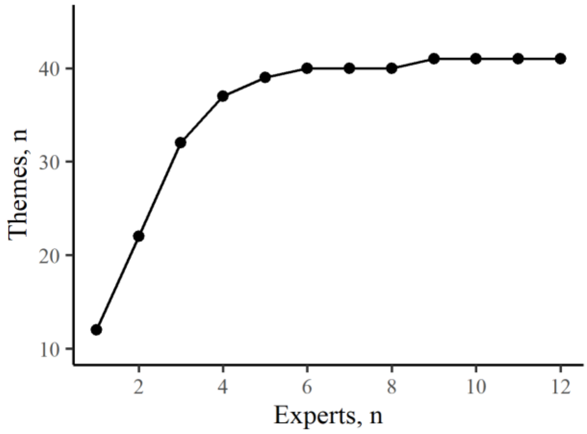

Figure 1. Number of new themes that was elicited during context analysis in order of expert inclusion
