# Supplementary material for: Expectations of Continuous Vital Signs Monitoring for Recognizing Complications After Esophagectomy: Interview Study Among Nurses and Surgeons
Source: JMIR Perioper Med. 2021 Feb 12;4(1):e22387. doi: 10.2196/22387 (PMC7910120; doi:10.2196/22387)
Supplement: Multimedia Appendix 4 [file periop_v4i1e22387_app4.pdf]

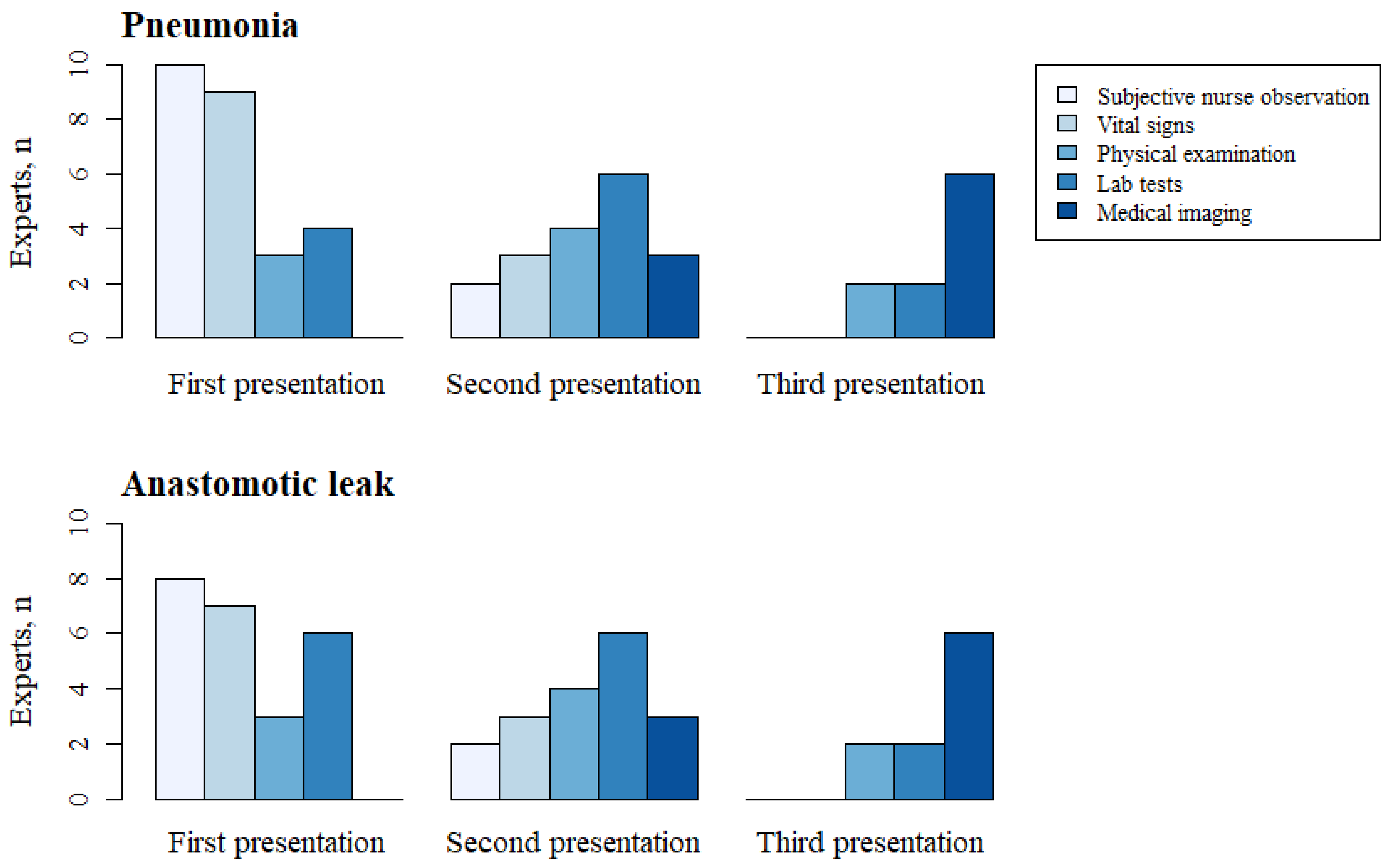

Typical order of early signs observed for pneumonia and anastomotic leak. Overview of the amount of experts (out of 12) that described that pneumonia or anastomotic leak is typically observed in the given routine measurement as first, second, or last sign of deterioration.
